# Novel Solid-State Emissive Polymers and Polymeric Blends from a T-Shaped Benzodifuran Scaffold: A Comparative Study

**DOI:** 10.3390/polym12030718

**Published:** 2020-03-24

**Authors:** Ugo Caruso, Rosita Diana, Angela Tuzi, Barbara Panunzi

**Affiliations:** 1 Department of Chemical Sciences, University of Napoli Federico II, 80126 Napoli, Italy; ugo.caruso@unina.it (U.C.); angela.tuzi@unina.it (A.T.); 2 Department of Agriculture, University of Napoli Federico II, 80055 Portici NA, Italy; barbara.panunzi@unina.it

**Keywords:** benzodifuran, polymer, fluorescence, photoluminescence quantum yield

## Abstract

Two novel polyimines were synthesized from a benzodifuran based diamino monomer and two dialdehydes bearing bulky groups and a flexible spacer. The polymers display tuned luminescence performance according to the presence of half-salen groups. The effect of the intramolecular bond on the emission properties were examined. Two model compounds, replicating the same emissive Schiff base cores, were synthetized. From the models, dye-doped blends in the fluorophore/matrix ratio, resembling the polymers, were produced. Amorphous thin films of the covalent polymers and the polymeric blends were obtained by spin-coating technique. The Photoluminescent (PL) response of the different macromolecular systems were qualitatively and quantitatively examined and compared.

## 1. Introduction

In recent years, solid-state highly emitting materials have gained significant attention due to the growing use of optoelectronic devices [1,2]. The wide range of potential applications in sensors [3,4], photo-modulators [5], luminescent switches [6], memory chips [7] and data storage technology [8] makes the class of solid-state emissive materials worthy of remarkable scientific and economic attention.

Organic materials, with extended donor-acceptor π-conjugated systems, featuring a large dipole moment in the excited state, are often reported as excellent candidates for all the optoelectronic applications [9]. The challenge in obtaining organic solid-state emissive materials can be overcome by the suitable assembly of macro-architectures. This mainly means dye-doped polymeric matrixes or covalently bonded emissive polymers. Covalently bonded polymers, obtained from low molecular monomers, often fulfil the requirements of processability, rheological stability and ease of synthesis. The flexibility of the organic chemistry allows the tailoring of the most varied π-conjugated structures with the required properties. Cost-effective, solubility, stability and processability must be added to the desired electronic and optical properties to obtain good optical quality thin films, readily integrable into devices. 

The factors governing solid-state emission of organic polymers are conjugation length, planarity and intramolecular interactions. The use of traditional fluorophores is limited due to aggregation-caused quenching (ACQ) effect [9,10,11,12]. The use of encumbered substituents is an effective strategy in preventing nonradiative decay channels, due to inter-chain interactions [13,14], and for suppressing dissipation pathways of the excited state energy. This effect is called restricted intramolecular rotation (RIR) effect [15]. For most of the molecules undergoing aggregation-induced emission (AIE) [16,17], the RIR effect is thought to be the dominant factor. Therefore, AIE-active molecules, with high solid-state emission, can be achieved by structural modifications and design strategies that alter macroscopic arrangements. 

**Scheme 1:**
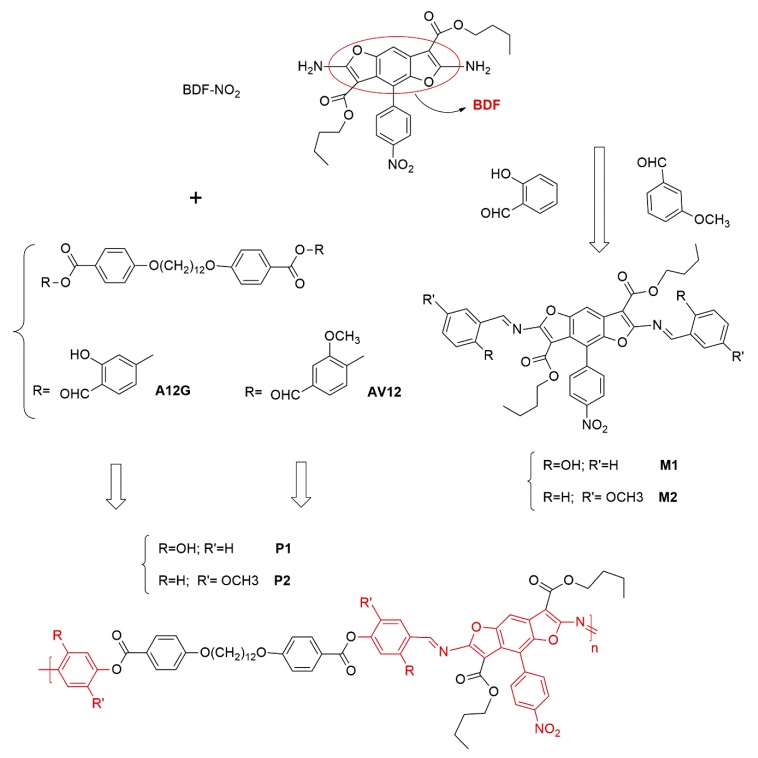
Synthetic route to polymers P1 and P2 and to models M1 and M2.

Benzodifuran (BDF, see Scheme 1) derivatives have received much attention in the biological and pharmacological areas [18,19], in the field of non-linear optical materials [20] and finally as building blocks in optoelectronic devices [21,22]. Due to the ease of modification and functionalization and to specific absorption–emission spectral properties, BDF scaffolds are an eclectic class of compounds, recently employed in optical waveguides, photovoltaic cells, OLEDs, and dye synthetized solar cells [21,23,24]. A symmetrical BDF core can be easily di-functionalized and employed as a monomer in the built of macro-architectures [25]. Insertion/modulation of the substituents on the BDF core can tune the chemical and optical properties.

In the present paper a planar diamino-BDF structure, bearing two alkoxy substituents and a *p*-nitrophenyl group (compound BDF-NO_2_ in Scheme 1) was employed to obtain different macromolecular solid-state emitters. In a previous article [19], a mechanistic study of the effect of the same BDF derivative on tumor cells was performed by means of fluorescence techniques, focusing interest on its photoluminescence (PL) properties in solution. Here we collected structural information on the difunctional BDF derivative by single crystals X-ray analysis. The donor-acceptor-donor (D–A–D) T-shaped architecture of BDF-NO_2_, with the nitro-phenyl group twisted with respect to the BDF core, potentially fulfils the requirement of RIR molecules. The monomer itself is not a luminophore in the solid state but its insertion in suitable macro-structures produced emissive materials. 

Two polyimines (P1 and P2 in Scheme 1) were synthesized from BDF-NO_2_ scaffold and two dialdehydes bearing a flexible spacer. The polymers display processability and tuned PL performance. The introduction in one case of half-salen groups led us to examine the effect of the intramolecular bond on the phase behavior and PL properties of the polymers. Two low-molecular weight model compounds (M1 and M2 in Scheme 1), featuring the basic emissive Schiff base core of the polymers, were synthetized and employed as dye-dopants in a host polymer. The models were dissolved in poly(vinylcarbazole) (PVK), a matrix typically employed in optical and optoelectronic devices [26,27]. The fluorophores were added to the host polymer in a similar percentage to the polyimines. PL performance of the covalent polymers and the polymeric blends was qualitatively and quantitatively examined and compared.

## 2. Materials and Methods 

### 2.1. Materials 

The compound A12G and AV12 [28] and the monomer dibutyl 2,6-diamino-4-(4-nitrophenyl)benzo[1–b:4,5-b′]difuran-3,7-dicarboxylate [19] BDF-NO_2_ were obtained as previously described. All commercially available products were supplied by Sigma Aldrich (St. Louis, MO, USA). Poly(9 vinylcarbazole) (PVK) was a molecular weight 1100 Da polymer.

### 2.2. Characterization

Optical observations were performed by using a Zeiss Axioscop polarizing microscope (Carl Zeiss, Oberkochen, Germany) equipped with an FP90 Mettler (Mettler-Toledo International INC MTD, Columbus, OH, USA) heating stage. Phase transition temperatures and enthalpies were measured using a DSC scanning calorimeter Perkin Elmer Pyris 1 (PerkinElmer, Inc., Waltham, MA, USA) at a scanning rate of 10 °C/min, under nitrogen flow. The decomposition temperature (*T*_d_) is assumed as the temperature where is recorded the 5 wt % weight loss. Decomposition temperatures (*T*_d_) were determined by thermogravimetric analysis under nitrogen flow, using a Perkin Elmer TGA 4000 (PerkinElmer, Inc., Waltham, MA, USA). UV-Visible and fluorescence spectra were recorded with JASCO spectrometers (JASCO Inc., Easton, MD, USA). Jasco F-530 (scan rate 200 nm min^−1,^ JASCO Inc., Easton, MD, USA) and on a spectrofluorometer Jasco FP-750 (excitation wavelengths set at the absorption maxima of the samples, scan rate 125 nm min^−1^, JASCO Inc., Easton, MD, USA). For the polymers P1 and P2 inherent viscosity (η_inh_) at 60.0 °C in 1,1,2,2 tetrachloroethane (TCE) was measured with an Ubbelohde viscometer. Mass spectrometry measurements were performed using a Q-TOF premier instrument (Waters, Milford, MA, USA) with an electrospray ion source and a hybrid quadrupole-time of flight analyzer. ^1^H NMR spectra were recorded in 1,1,2,2-tetrachlorethane-d2 with a Bruker Advance II 400MHz apparatus (Bruker Corporation, Billerica, MA, USA).

### 2.3. Preparation of Thin Films for Optical Characterizations and PLQY Setup

All thin film samples used for photoluminescence quantum yield (PLQY) characterizations were made by an SCS P6700 spin-coater apparatus (Specialty Coating Systems Inc., Indianapolis, IN, USA). Both polymers and blend samples (50% by weight of M1 or M2 in PVK) dissolved in 20mg/mL 1,1,2,2 tetrachloroethane (TCE) were deposed on quartz slides by the spin-coating technique operating at 600 rpm for 60′ and annealed at 100 °C for 10′.

Photoluminescence quantum efficiency values were recorded on quartz slides by a Fluorolog 3 spectrofluorometer (Horiba Jobin Instruments SA, Edison, NJ, USA), within an integration sphere equipped with a fiber optic connection.

### 2.4. X-ray Analysis

Single crystals of BDF-NO_2_ appropriate for X-ray crystal structure analysis were obtained from gradual evaporation of toluene/heptane solution at room temperature. A crystal was installed under nitrogen flux at 173 K on a Bruker-Nonius KappaCCD diffractometer fitted out with 700 Oxford Cryostream apparatus (graphite monochromated MoK_α_ radiation, λ = 0.71073 Å, CCD rotation images, thick slices, *φ* and *ω* scans to fill asymmetric unit). Semi-empirical absorption corrections have been applied (SADABS [29]). Direct methods have been utilized to solve the structure (SIR97 program [30]) and anisotropically perfected with the full matrix least squares method on *F*^2^ against all independent measured reflections by the use of SHELXL-2018/3 (Sheldrick, 2018, Dept. of Structural Chemistry, Göttingen, Germany) [31] and WinGX software (Louis J. Farrugia, University Of Glasgow, Glasgow, United Kingdom) [32]. The H atoms at amine groups were found in difference electron-density maps and freely refined with *U*_iso_(H) equal to 1.2*U*_eq_ of the carrier atom. All the other H atoms were generated stereochemically and were refined by the riding model [C–H = 0.93–0.96 Å]. The remaining hydrogen atoms were introduced into estimated points and refined matching to a driving model with distances with C–H distances in the range 0.95–0.99 Å and *U*_iso_(H) equal to 1.2*U*_eq_ of the carrier atom (1.5 *U*_eq_ C_methyl_). For all H atoms Uiso(H) = 1.2Ueq(C) or 1.5Ueq(C–methyl). The butyl group bound at O5 is disordered over two positions with refined occupancy factors of 0.562(9) and 0.438(9). Some constraints were introduced at the last stage of refinement using a distance geometrical restraint commands DFIX and SIMU commands of SHELXL program. All crystallographic data and structure refinement details are reported in Table 1. Hydrogen bonds geometry is reported in Table 2. The figures were generated using ORTEP-3 [33] and Mercury CSD 4.0 [34] programs.

### 2.5. Synthesis of the Polymers

All polymers were obtained in solution following the same synthetic route. We described, as example, the synthesis of polymer P1. A total of 0.683 g (1.00 mmol) of dialdehyde A12G and 0.509 g (1.00 mmol) of diamine BDF-NO_2_ were dissolved in 4 mL of tetrachloroethane (TCE) with 0.5 mL of glacial acetic acid under a nitrogen atmosphere. The reaction proceeded at boiling temperature for 45′, then the solution was poured into hexane and the polymer obtained by precipitation was cleaned in methanol twice and dried in an oven at 80 °C. Yields were about 70% in all cases. ^1^H NMR (400 MHz, TCE-d2, 25 °C, ppm): 1.32 (6H), 1.47 (20H), 1.82 (8H), 3.99 (4H), 4.56 (4H), 6.56 (0.58H), 7.02 (4H), 7.50 (5H), 7.77 (2H), 8.20 (2H), 8.42 (2H), 8.82 (4H), 10.00 (2H), 13.05 (2H).

P2: ^1^H NMR (400 MHz, TCE-d2, 25 °C, ppm): 1.29 (6H), 1.49 (20H), 1.78 (8H), 3.92 (6H), 4.03 (4H), 4.54 (4H), 6.56 (0.48), 7.00 (4H), 7.47 (4H), 7.42 (2H), 7.68 (4H), 8.45 (2H), 8.78 (3H), 9.97 (2H).

### 2.6. Synthesis of the Models

The synthesis of the model compounds was performed in solution following the same basic procedure for both samples. Description is reported for M1. A total of 0.509 g (1.00 mmol) of BDF-NO_2_ and 0.488 g (4.00 mmol) of 2-hydroxybenzaldehyde was dissolved in 8 mL of glacial acetic acid, and the reaction was carried out for 30 min at boiling temperature. The solution is then cooled at room temperature the formation of a red precipitate ensuing. Yield = 43%. ^1^H NMR (400 MHz, TCE-d2, 25 °C, ppm): 1.32 (t, 6H), 1.47 (m, 4H), 1.83 (m, 4H), 4.61 (t, 4H), 7.02 (dd, 4H), 7.32 (m, 2H), 7.53 (m, 3H), 8.15 (d, 4H), 10.16 (s, 2H).

Elemental analysis calculated (%) for C_41_H_37_N_3_O_10_: C, 67.30; H, 5.10; N, 5.74; found: C, 67.40; H, 4.99; N, 5.78. MALDI-TOF of A1 m/z: 732.27 (M + H).

M2: ^1^H NMR (400 MHz, TCE-d2, 25 °C, ppm): 1.30 (t, 6H), 1.49 (m, 4H), 1.83 (m, 4H), 3.92 (s, 3H), 4.30 (t, 4H), 7.00 (dd, 4H), 7.30 (m, 2H), 7.58 (m, 3H), 8.27 (d, 4H), 10.07 (s, 2H).

Elemental analysis calculated (%) for C_42_H_39_N_3_O_10_: C, 67.64; H, 5.27; N, 5.63; found: C, 67.50; H, 5.29; N, 5.70. MALDI-TOF of A1 m/z: 746.79 (M + H).

## 3. Results and Discussion

### 3.1. Polymers and Model Compounds

As summarized in Scheme 1, the polymers P1 and P2 were obtained by condensation of the diamino derivative BDF-NO_2_ with dialdehydes A12G, or AV12, respectively. The flexible (CH_2_)_12_ spacer of the dialdehydes and the butoxy groups of BDF guaranteed solubility and processability. Both polymers resulted soluble in the organic solvents as TCE, N-methyl pyrrolidinone (NMP), dimethylformamide, dimethyl sulfoxide, o-dichlorobenzene and chemically stable as solid materials above 300 in air (TGA data). In order to keep the material processable, the reaction was stopped after 45′. For longer reaction time, the products obtained were slightly insoluble.

As the emissive cores in P1 and P2 formed upon polycondensation, the basic fluorogenic units of the polymers are respectively M1 and M2 (see Scheme 1, correspondent to the part marked in red of the polymers). The models were obtained by using glacial acetic acid as a solvent. The best synthetic results were obtained using a large stoichiometric excess of the aldehydes. The butyl chains increasing solubility led to compounds highly processable. 

The crystalline models are respectively red and yellow emitters (CIE (Commission Internationale de l’Eclairage) coordinates are reported in Table 3). PLQYs (photoluminescent quantum yields) were recorded on samples deposed on quartz slides by spin-coating. In the solid phase M1 crystals reaches 23% PLQY, the result for a red emitter with the maximum above 600 nm, CIE coordinates (0.40; 0.37) were interesting. Conversely, M2 bearing a methoxy group in *meta* position respect to the imine bond, is a scarcely emissive yellow fluorophore (see Table 3). 

The T-shaped structure of the models involves a D–A–D pattern where the strong electron-withdrawing moiety is the *p*-nitrophenyl substituent. Although structurally similar, the relevant difference between the two emissive fragments is the phenolic group, leading to intramolecular hydrogen-bond interactions in the *half-salen* moieties. Due to the torsional hindrance due to H-bonds, the aromatic main plane is extended and stiffened in M1. The T-shaped pattern, between the main plane and the nitrophenyl ring, is locked. This finding proves that the same electron-rich building blocks in the D–A–D pattern undergo non-radiative deactivation processes in absence of the torsional barrier. 

**Figure 1:**
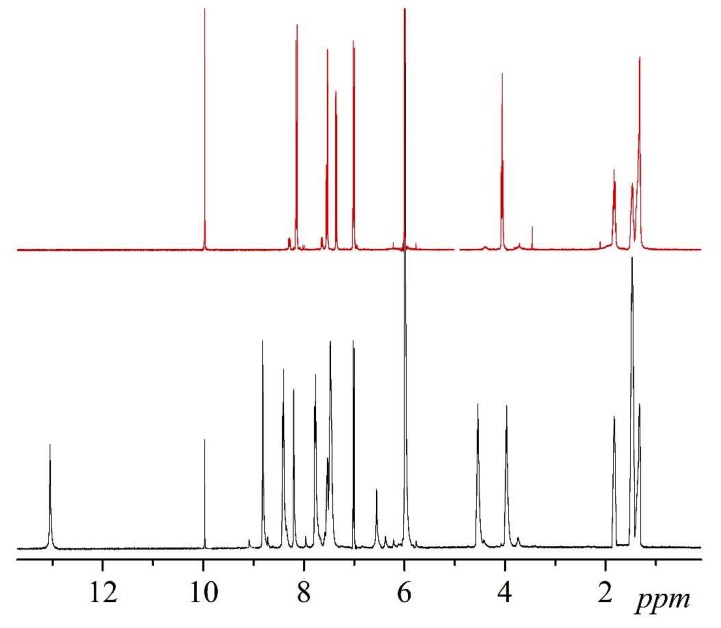
^1^H-NMR spectrum of P1 (**black curve**) and the correspondent model M1 (**red curve**).

In Figure 1 and in Table 4 (most relevant ^1^H-NMR resonances) a comparison between NMR protonic spectra of M1 (red curve) and the related polymer P1 (black curve), is shown. Both in P1 and M1 the imine signal is recorded at about 10 ppm, which is higher than a typical CH=N signal. With respect to the model, the aromatic pattern of the polymer is complicated by the addition of the benzoyloxy groups of A12G monomer. At 4.56 and 3.99 ppm the OCH_2_ protons of the diamine and of the dialdehyde, respectively, were detected. At 13.05 ppm the phenolic hydrogens, not recorded in M1 spectrum, is observable for P1. Based on the NMR integration of the monomeric units and the terminal groups (NH_2_ at 6.56 ppm), the molecular weight can be estimated around 4000 Da. This means three/four repetitive units, justifying quite sharp signals in NMR pattern of P1. Similar considerations can be done for P2. Intrinsic viscosity measured for the polymers reacted for 45′ is 1.05 and 0.95 dL g^−l^ respectively. 

**Figure 2:**
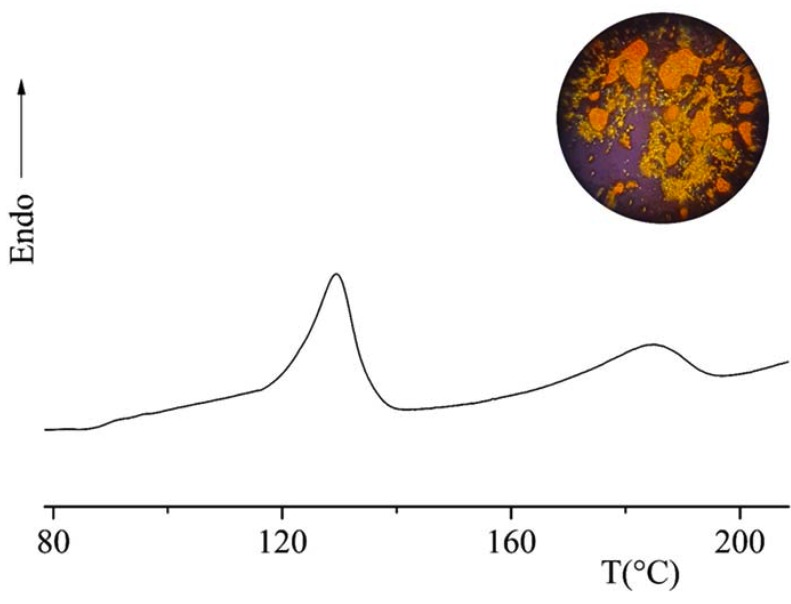
DSC thermogram of P1 and its nematic schlieren texture (inset) photographed under irradiation by UV-visible lamp at 365 nm.

The polymers as precipitated from the reaction mixture show some extent of crystallinity, as observed by optical microscopy and confirmed by X-ray diffraction spectrum of powder samples. The crystallinity is probably due to some constitutional order characterizing the sequence of the aromatic groups along the main chain. Compound P1 undergoes glass transition with melting of the crystalline amount at 129 °C. After melting the compound is a nematic liquid with isotropization at 185 °C. The LC behavior due to the alternation of rigid elongated groups and flexible chains is not unexpected on the base of our previous results [28]. The DSC curve of a sample of P1 is shown in Figure 2. The signals related to the glass transition overlapped to the melting of the crystalline part (peaked at 129 °C), and the endotherm signal at 185 °C due to the isotropization are clearly observable. DSC of polymer P2 shows analogous pattern with a lower stability range of the nematic phase (see Table 5). The better ability of P1 to retain the mesophase can be ascribed to the H-bond interactions, able to produce a wider aromatic main plane. 

In both cases the nematic schlieren texture of the polymers is luminescent under UV-visible lamp at 365 nm (see Figure 2, inset).

A qualitative and quantitative analysis was performed on the emission pattern of the polymers. In solution polymers are poor emitters. For solid-state PL measurements polymers were dissolved in TCE and spin-coated onto quartz slides. The semi-crystalline habitus obtained recovering the product by slow precipitation was lost after spin-coating. The fast quenching of the polymeric film during the spin-coating process led to homogeneous and transparent thin layers. The film samples resulted unaltered up to three months. For application, the amorphous status could be an advantage. As their PL performance is independent of the crystalline order, attention should not be paid to the deposition methods [35,36].

The film samples of P1 and P2 show very different behavior. A relevant fluorescence was displayed by P1 in the solid state, with medium PLQY, large Stokes shift and CIE coordinates in the red region (see Table 3). Conversely, polymer P2 shows scarce emission peaked in the yellow region (Table 3). 

As mentioned, in both polymers the luminescent core consists of the fragment depicted in red in Scheme 1. In addition, blocks can be added to the polymer chain to improve PL performance. The benzoyl bulky groups of the dialdehydes A12G and AV12 were added to restrict chain motion and reduce detrimental intermolecular interactions [14,37,38]. 

As in the model compounds, the crucial difference between the two materials is the H-bond. The aromatic main plane of the fluorophore core in presence of the intramolecular H-bond is locked in a longer and stiffer conjugated system forming a T with the twisted nitrophenyl moiety. Such arrangement, hardly subjected to rotation, is frozen in the polymeric chains making P1 a better RIR candidate, with higher PLQY. As the organic materials rarely show efficient red emissions due to the high degree of self-quenching for the large dipole moments (energy gap law [39,40,41]) P1 can be considered an interesting red polymeric luminophore. 

As expected, PL performance of polymers and related models show analogies. The red-shift in the emission maxima respect to the absorption ones for all systems is not unexpected [42,43]. Nevertheless, PLQYs measured on the crystalline models cannot be directly compared with the amorphous polymeric sample.

### 3.2. PVK Blends

In our attempt to improve the PL performance of low-molecular weight emitters we previously employed the technique to dissolve/disperse the fluorophores in polymeric matrices. As a matrix typically employed in OLEDs (Organic Light Emitting Diode) construction [44] PVK is a conductive amorphous scarcely emissive and highly processable host polymer. The dye-doped blends can be a way for some chromophore to relieve ACQ effect [11], because the system resemble a diluted solid solution of the emitters frozen in an amorphous medium. In the present case, the analysis of the emission properties was carried out on PVK blends of the active units M1 and M2. The doped blends were obtained by dissolving in TCE both PVK matrix and the dye, in the ratio reproducing the covalent polymers. Due to the solubility limit of the dyes in the PVK matrix, the real 60% wt. ratio cannot be reached, and the examined samples were 55 wt % blends. Transparent amorphous thin films were produced by spin-coating the blends.

**Figure 3:**
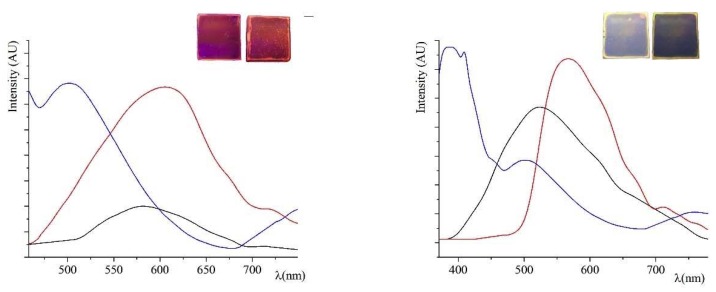
Normalized emission curves recorded on polymers and 50% PVK blends. On the left: Thin film of P1 (red curve), M1-PVK (black curve) and PVK (blue curve) as reference. On the right: P2 (red curve), M2-PVK (black curve) and PVK (blue curve) as reference. The same spin-coated thin films photographed under the UV lamp at 365 nm in the inset (polymer on the **left** and blend on the **right**).

In Figure 3, the emission spectra of the polymers P1 and P2 are compared with the related blends M1-PVK and M2-PVK. The intrinsic emission ability of M1 and M2 fluorophores reflected in the two macromolecular amorphous systems. The similarity in the spectroscopic pattern, Stokes shifts and CIE coordinates are mostly respected. P1 and M1-PVK show emission maxima in the red region. The PL performance of M1 moiety, in both materials, is a remarkable achievement for a red fluorophore. Conversely, P2 and M2-PVK are poor yellow emitters and the emission maxima differ by 43 nm. Due to the scarce emission of the fluorophore, a contribution of the PVK matrix to the emission of M2 blend becomes heavy. Not unexpectedly, the production of PVK blends causes a slight shift toward shorter wavelength region of the emission maxima of M1 and M2. The effect is due to the contribution of the blue emission of the polymer host. Conversely, the absorption is not much affected because PVK is a colorless matrix.

In both cases, PLQYs of the polymers are higher than blends, validating the practice of covalently bond the fluorophores in a polymeric chain where suitable bulky blocks can amplify the RIR effect.

### 3.3. Single Crystal X-ray Analysis of BDF–NO_2_

The main building block in the polymers and in the related monomers is BDF-NO_2_. Due to the alkyl ester substituents and the tilted *p*-nitrophenyl ring this fragment potentially undergoes RIR effect if included in a suitable chemical environment.

**Figure 4:**
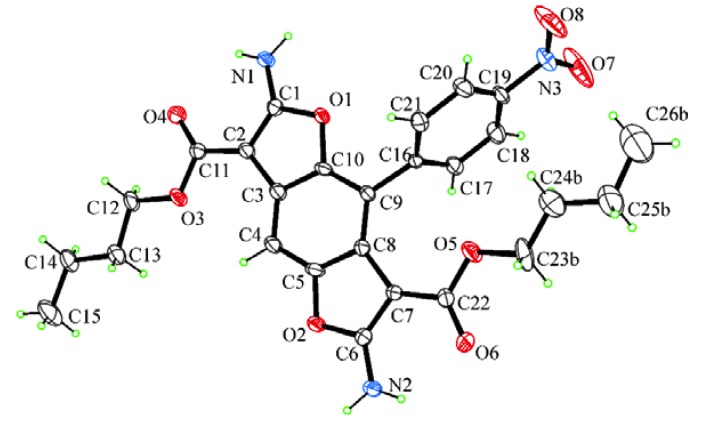
ORTEP view of BDF-NO_2_ with thermal ellipsoids drawn at 50% probability level. Only the major part of the disordered butyl group is drawn for clarity.

The molecular structure of BDF-NO_2_ is shown in Figure 4. Compound crystallizes in the monoclinic C2/c space group with one molecule contained in the asymmetric unit. Crystal data and structure refinement details are reported in Table 1. All bond lengths and angles are in expected range and in agreement with analogous compounds [25,45].

**Figure 5:**
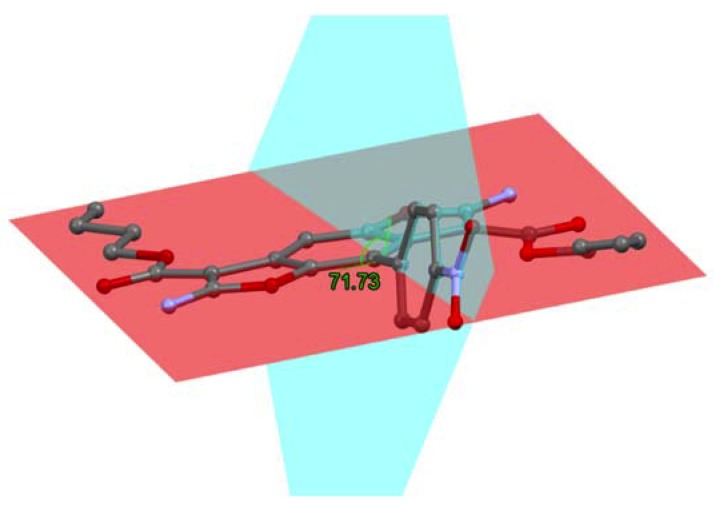
Angle between mean the benzofurane (red plane) and the nitrobenzene (light blue plane) mean planes. H atoms are not drawn for clarity.

In the molecule the intramolecular hydrogen bonds between amine donor groups and carbonyl oxygen atoms enforce ester groups to be planar with benzodifuran ring. The nitrophenyl ring is highly twisted with respect to benzodifuran to minimize interactions with the close bulky butyl-ester group (Figure 5). Due to the strong intramolecular NH···O=C hydrogen bond that lock the ester group in the observed conformation, a hindering to rotation of nitrobenzene ring may be expected on the excited state. Such hindered rotation satisfies the requirements of RIR molecules. A planar geometry at N amine atoms and a shortening of C1–N1 and C6–N2 bond distances (1.328(3) and 1.320(3)Å) suggest a partial conjugation of N amine atom with benzofuran ring, according with a push-pull (D–A–D) T-shaped system. 

**Figure 6:**
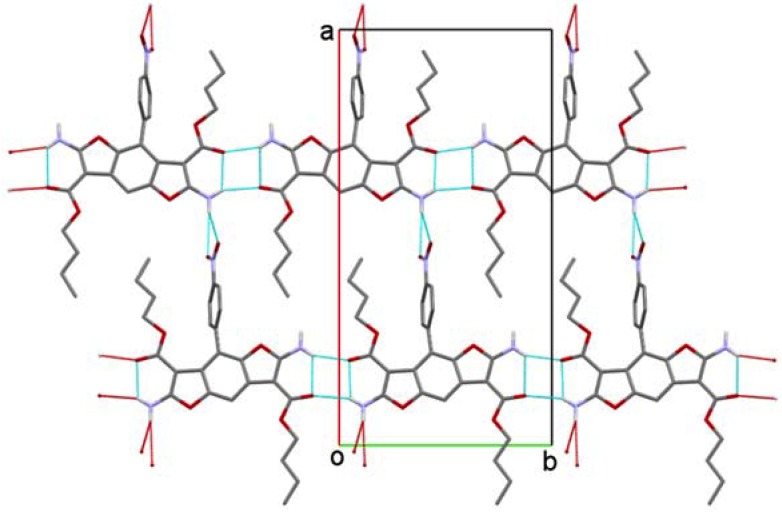
Partial packing showing a sheet of H-bonded molecules viewed along c axis direction. Only the amine hydrogen atoms are shown for clarity. Hydrogen bonds are drawn as cyan lines (red lines are for pending contacts).

**Figure 7:**
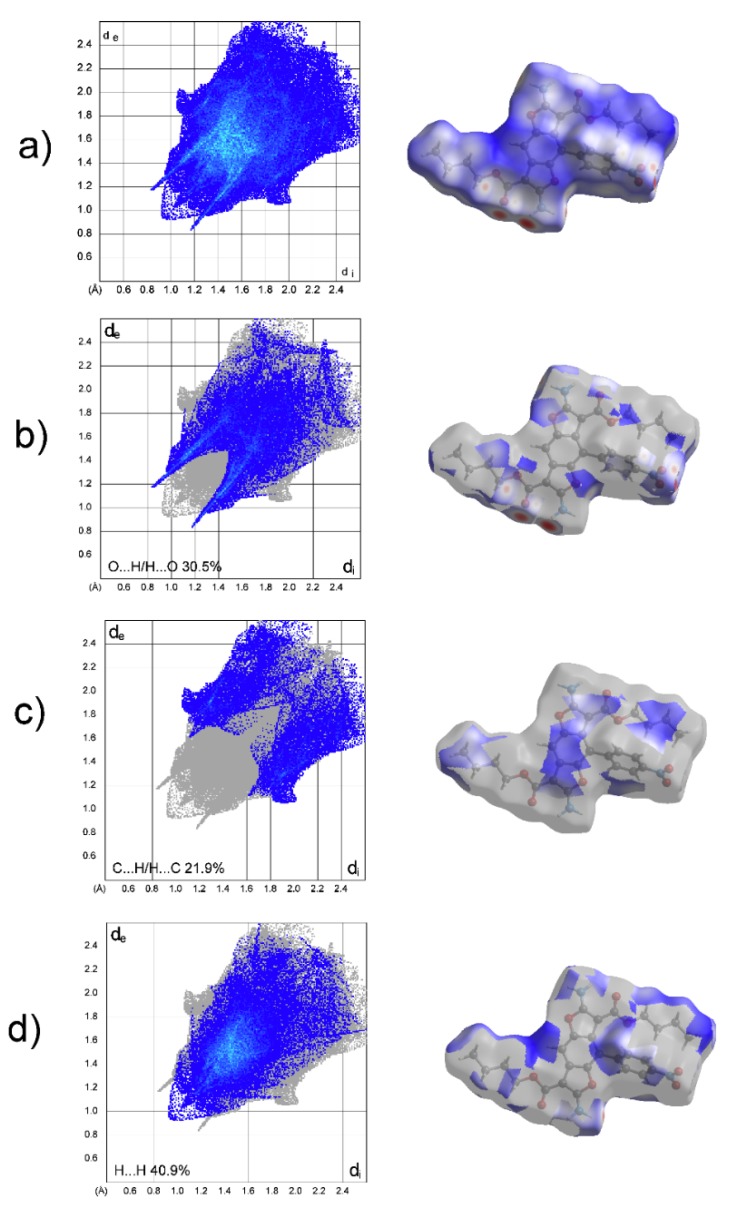
Two-dimensional fingerprint plots of BDF-NO_2_ (LEFT) and the corresponding d_norm_ Hirshfeld surfaces (RIGHT). (**a**) Total fingerprint plot; (**b**), (**c**), (**d**) fingerprint plots of most relevant intermolecular interactions. The red dots on the d_norm_ surfaces are diagnostic of hydrogen bonds.

The crystal packing is dominated by strong N–H···O hydrogen bonds and weak C–H···O interactions (Table 2). The donor amine groups are involved in intermolecular H-bonds with the acceptor carbonyl and nitro groups forming sheets of molecules piled up in the **c** axis direction (Figure 6). Molecules self-assemble into a two-dimensional hydrogen bonded network with interdigitate alkyl-alkyl and alkyl-nitrophenyl interactions. No twisting of the nitro group is observed in the solid state and the presence of the intermolecular hydrogen bonds between the donor -NH_2_ and the acceptor –NO_2_ groups could restrict the rotation of nitro group on the excited state [46,47]. No π···π interactions are found, as confirmed by Hirshfeld surfaces analysis and the two-dimensional fingerprint plots (Figure 7), and calculated using *CrystalExplorer17.5* program [48].

Crystal data were deposited at Cambridge Crystallographic Data Centre with assigned number CCDC 1986514. These data can be obtained free of charge from www.ccdc.cam.ac.uk/data_request/cif.

## 4. Conclusions

We examined the PL performance of a diamino functionalized T-shaped BDF scaffold in different macromolecular systems. Through X-ray crystallography, we found that the T-shaped D–A–D pattern of the monomer potentially fulfils the requirement of RIR molecules. By reacting BDF–NO_2_ with two dialdehydes, bearing bulky groups and a flexible spacer, we obtained two nematic polymers. The polymers are soluble, stable and processable into amorphous thin films, with red and yellow emission in the amorphous solid state. The basic emissive cores of the polymers were reproduced into two model compounds used to compare the PL performance of the covalent polymers with the related dye-doped PVK blends. The different macro-structures show similar emission behaviour. Both P1 and its blend are red emitters with noteworthy PLQY, due to the RIR undergoing half-salen emissive cores. Conversely, P2 and the related blend are poor yellow emitters. In both cases, the blends show PLQYs lower than the covalent polymers. This is an interesting achievement. Compared with the doped macromolecules, covalent polymers better satisfy the requirements for devices, such as processability, rheological stability, reproducibility and tailoring of synthesis.

## Figures and Tables

**Table 1:** Crystal data and structure refinement details for NO_2_C_4_.

CCDC Number	1986514
Empirical Formula	C_26_H_27_N_3_O_8_
Formula Weight	509.50
Temperature (K)	173(2)
Wavelength (Å)	0.71073
Crystal System (Å)	Monoclinic
Space Group	*C2*/*c*
*a* (Å)	25.644(3)
*b* (Å)	12.1350(16)
*c* (Å)	16.8623(16)
*α* (°)	90
*β* (°)	112.021(7)
*γ* (°)	90
Volume (Å^3^)	4864.6(10)
Z	8
D_calc_ (Mg/m^3^)	1.391
*μ* (mm^−1^)	0.104
F(000)	2144
Crystal Size (mm)	0.50 × 0.25 × 0.10
*θ* Range (°)	2.935 to 27.495
Reflections Collected/Unique	17016/5461 [R(int) = 0.0605]
Refinement Method	Full-matrix least-squares on *F^2^*
Data/Restraints/Parameters	5461/103/386
Goodness-of-fit on *F*^2^	1.038
Final *R* Indices [I > 2 sigma(I)]	*R1* = 0.0633, *wR2* = 0.1304
*R* Indices (all data)	*R1* = 0.1331, *wR2* = 0.1583
Largest diff. Peak and Hole (eA^−3^)	0.638 and −0.385

**Table 2:** Hydrogen bonds for NO_2_C_4_ [Å and °].

D–H…A	d(D–H)	d(H…A)	d(D…A)	<(DHA)
C20–H15…O8*^i^*	0.95	2.66	3.566(4)	160.5
C20–H15…O8*^i^*	0.95	2.66	3.566(4)	160.5
C12–H18A…O7*^ii^*	0.99	2.54	3.356(4)	139.2
N1–H1B…O4	0.82(3)	2.38(3)	2.917(3)	123(2)
N1–H1B…O6*^iii^*	0.82(3)	2.19(3)	2.902(3)	146(3)
N2–H2A…O7*^iv^*	0.86(3)	2.57(3)	3.378(4)	156(3)
N2–H2A…O8*^iv^*	0.86(3)	2.42(3)	3.213(3)	154(3)
N2–H2B…O4*^v^*	0.85(3)	2.32(3)	3.059(3)	145(3)
N2–H2B…O6	0.85(3)	2.22(3)	2.777(3)	124(3)

Symmetry transformations used to generate equivalent atoms: *i* = −x + 2, y, −z + 3/2; *ii* = −x + 3/2, y − 1/2, −z + 3/2; *iii* = x, y − 1, z; *iv* = x − 1/2, −y + 1/2, z − 1/2; *v* = x, y + 1, z.

**Table 3:** Optical data of models, polymers and blends.

Sample	λ_ab.sol_ (nm)^[a]^	λ_em.sol_ (nm)^[b]^	λ_ab.film_ (nm)^[c]^	λ_em.film_ (nm)^[d]^	CIE Coord.^[e]^	Stokes Shift (nm)^[f]^	PLQY%^[g]^
**M1**	456	528	470	607	0.40; 0.37	137	23 ± 0.2
**M2**	430	504	430	550	0.39; 0.46	120	1.5 ± 0.2
**P1**	457	512	453	609	0.42; 0.33	156	22 ± 0.2
**P2**	438	515	435	565	0.36; 0.41	130	2.0 ± 0.2
**M1-PVK**	-	-	443	582	0.40; 0.35	139	14 ± 0.2
**M2-PVK**	-	-	411	522	0.39; 0.45	111	1.5 ± 0.2

[a] Wavelength of UV-Visible absorbance maxima in NMP solution; [b] wavelength of emission maxima in NMP solution (excitation wavelength 400 nm); [c] wavelength of UV-Visible absorbance maxima on spin-coated thin films; [d] wavelength of emission maxima on spin-coated thin films (excited to absorption maximum wavelength); [e] emission CIE coordinates [f] Stokes shift (from emission to absorption); [g] PL quantum yield measured on thin films.

**Table 4:** Most relevant ^1^H-NMR resonances.

Group	Chemical Shift (ppm)			
	M1	M2	P1	P2
OCH_3_		3.92		3.92
OCH_2_ (of dialdehyde)	3.99	4.03		
OCH_2_ (of BDF-NO_2_)	4.56	4.54	4.61	4.30
NH_2_	6.56	6.56		
CN=N	10.00	9.97	10.16	10.07
OH	13.05			

**Table 5:** Thermal data of models and polymers.

Compound	*M*p/*T*_g_ (°C)^[a]^	*T*_i_ (°C)^[b]^	*T*_d_ (°C)^[c]^
**M1**	261	-	288
**M2**	289	-	296
**P1**	129	188	330
**P2**	120	130	325

[a] Melting point or glass transition temperature; [b] isotropization temperature; [c] Decomposition temperature.
